# A case report of a patient with stage IIIa hepatocellular carcinoma complicated by early gastric cancer

**DOI:** 10.3389/fonc.2024.1440171

**Published:** 2024-11-25

**Authors:** Xiaojing Song, Rangji Cai, Lili Wang, Fanghui Ding, Haixu Ni

**Affiliations:** ^1^ General Surgery Department of Lanzhou University First Hospital, No.1, Chengguan, Lanzhou, Gansu, China; ^2^ Internal Medicine Department, Luqu County Tibetan Hospital, Luqu County, Gansu, China; ^3^ Imaging Department of Lanzhou University First Hospital, No.1, Chengguan, Lanzhou, Gansu, China

**Keywords:** liver cancer, targeted therapy, radiotherapy, early gastric cancer, stage IIIA

## Abstract

We report a case of patient with Stage IIIa Hepatocellular Carcinoma Complicated by Early Gastric Cancer. Although Stage IIIa liver cancer can be treated with surgery, the overall prognosis of surgery is not ideal. Alternatively, conversion therapy is reported with different effectiveness towards stage IIIa liver cancer. Herein, this study shared the successful conversion of a patient with stage IIIa liver cancer and with early gastric cancer at Lanzhou University First Hospital, which is hoped to engage clinicians in evaluation and discussion.

## 1 Introduction

Primary liver cancer (PLC) accounts for 8.2% of all cancer deaths worldwide, and is the fourth most lethal malignancy (1, 2). There are 782,000 deaths and 841,000 new cases of PLC worldwide every year, with East Asia being the region with the highest prevalence of the disease (1). Hepatocellular carcinoma (HCC) constitutes for 85-90% of primary liver malignancies, and is the fourth most prevalent and the second most deadly cancer in China (3).

Barcelona clinic liver cancer (BCLC) classification has been widely validated. Due to variability of HCC prevalence, phenotype, and treatment response in China, specific guidelines for Chinese patients have been established (4). China liver cancer staging (CNLC) system was found in 2017, and has been used since then. In CNLC system, each stage of BCLC 0/A, B, and C is divided into two sub-stages, including stages Ia, Ib, IIa, IIb, IIIa, and IIIb (5).

For CNLC stage IIIa primary liver cancer, most patients have no indication for surgical resection (6), and for those with good liver function, the Chinese liver cancer staging system recommends TACE as the preferred treatment (5), in addition to systemic anti-tumor therapy (chemotherapy, targeted and immunotherapy) and radiotherapy (internal and external radiotherapy), which can be applied alone or in combination. The combination of TACE with targeted and immunotherapy treatment has significantly prolonged progression-free survival and overall survival (7–10). However, the outcomes remain still limited. In the present study, we report on a case, in which complicated by early gastric cancer. We hope this case will help clinicians to improve the treatment strategy for HCC.

## 2 Case report

**Figure 1:**
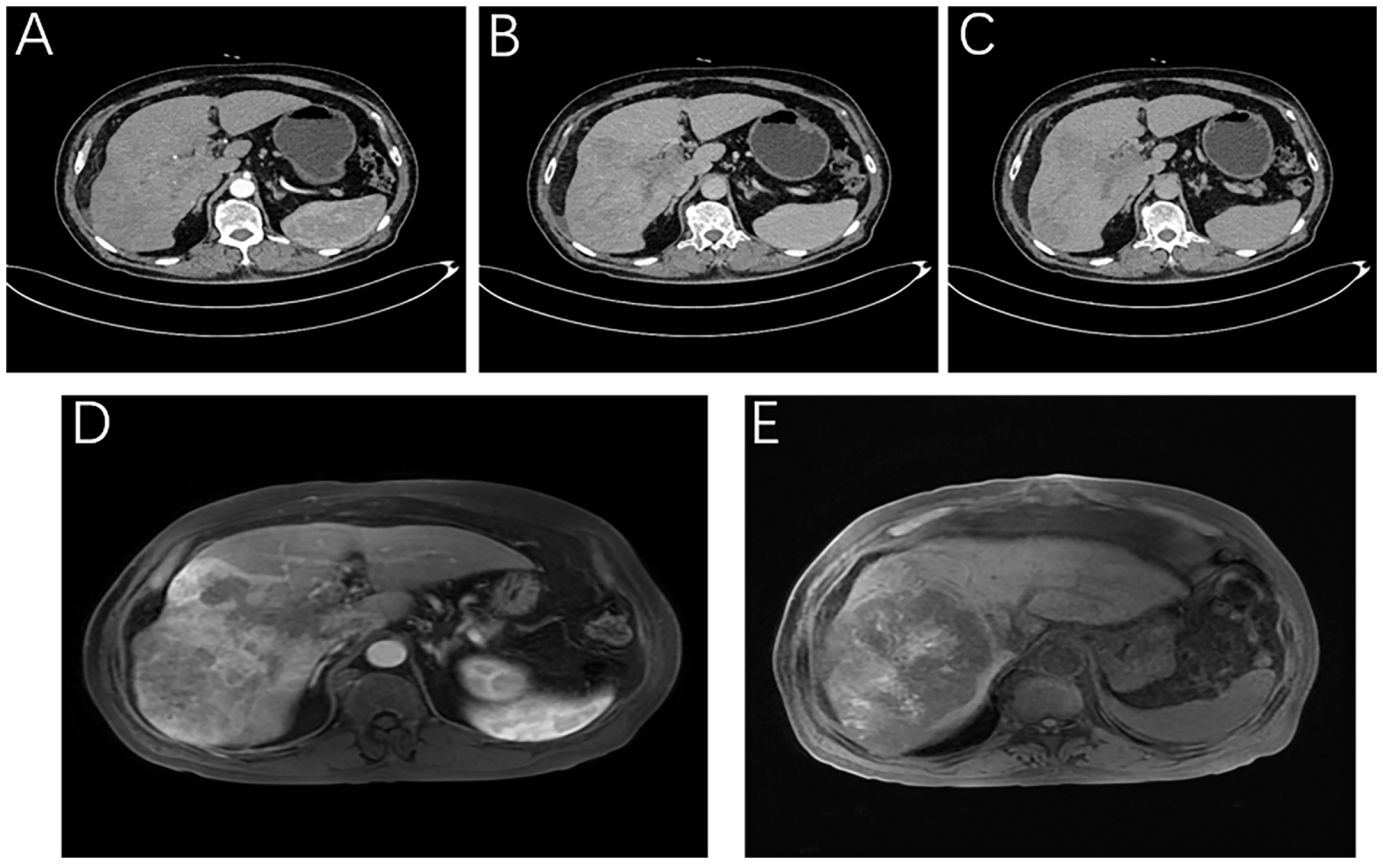
Imaging of the patient at the time of initial diagnosis. Liver-specific MRI: **(A)** Arterial phase, **(B)** Portal vein stage (blood vessel), **(C)** Grace; Enhanced abdominal CT: **(D)** Portal stage: portal vein thrombosis, **(E)** T1WI intratumor hemorrhage.

**Figure 2:**
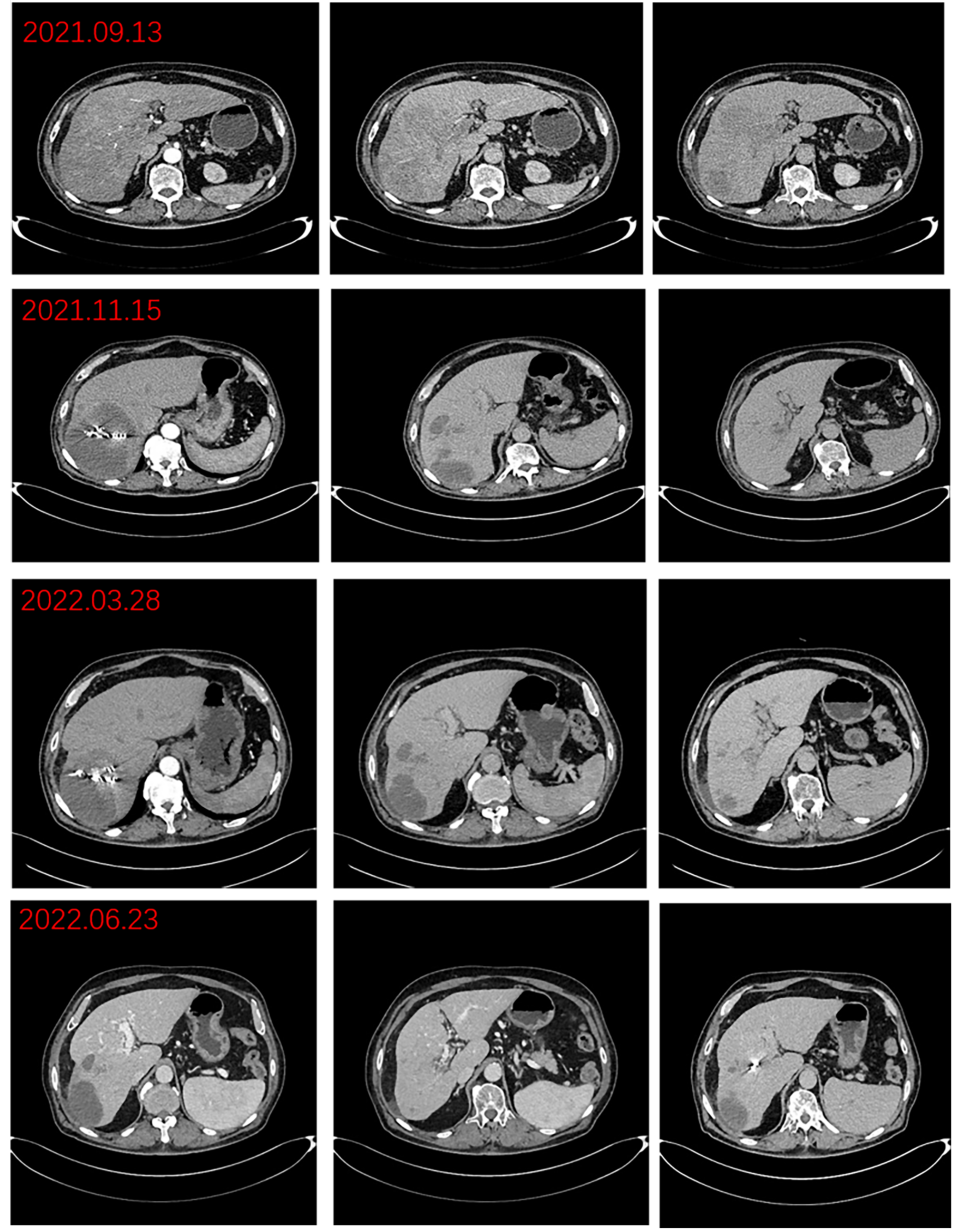
Preoperative imaging changes of hepatocellular carcinoma lesions.

**Figure 3:**
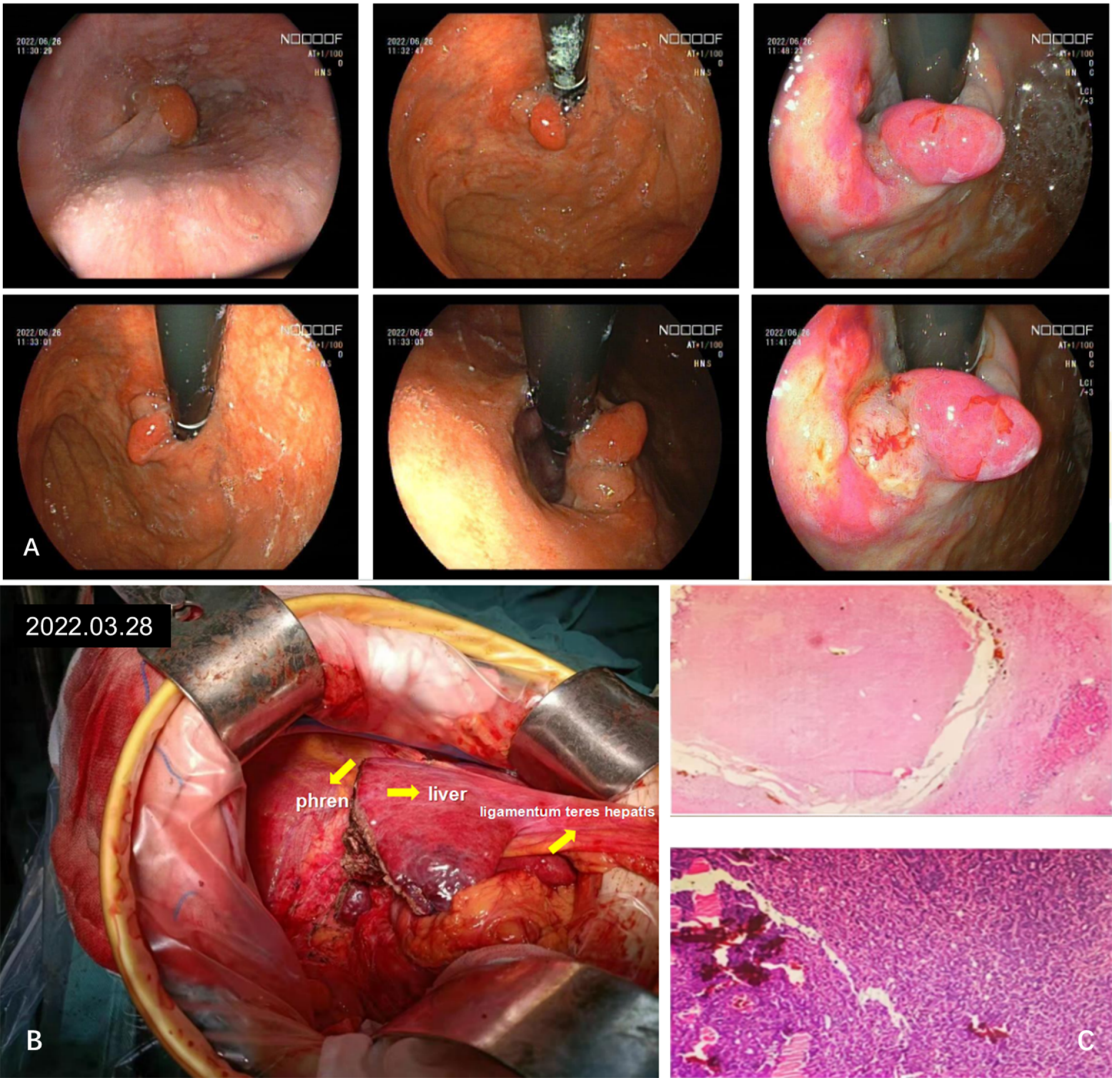
Diagnosis and intraoperative imaging of hepatocellular carcinoma combined with early gastric cancer. **(A)** A gastroscopic lesion of type 0-Ia+IIc is observed near the anterior wall of the cardia. **(B)** Intraoperative image. **(C)** Extensive sampling of liver tissue revealed all necrotic tissue, while gastric tissue exhibited chronic inflammation and focal low-grade intraepithelial tumors, as observed by hematoxylin-eosin (HE) staining.

**Figure 4:**
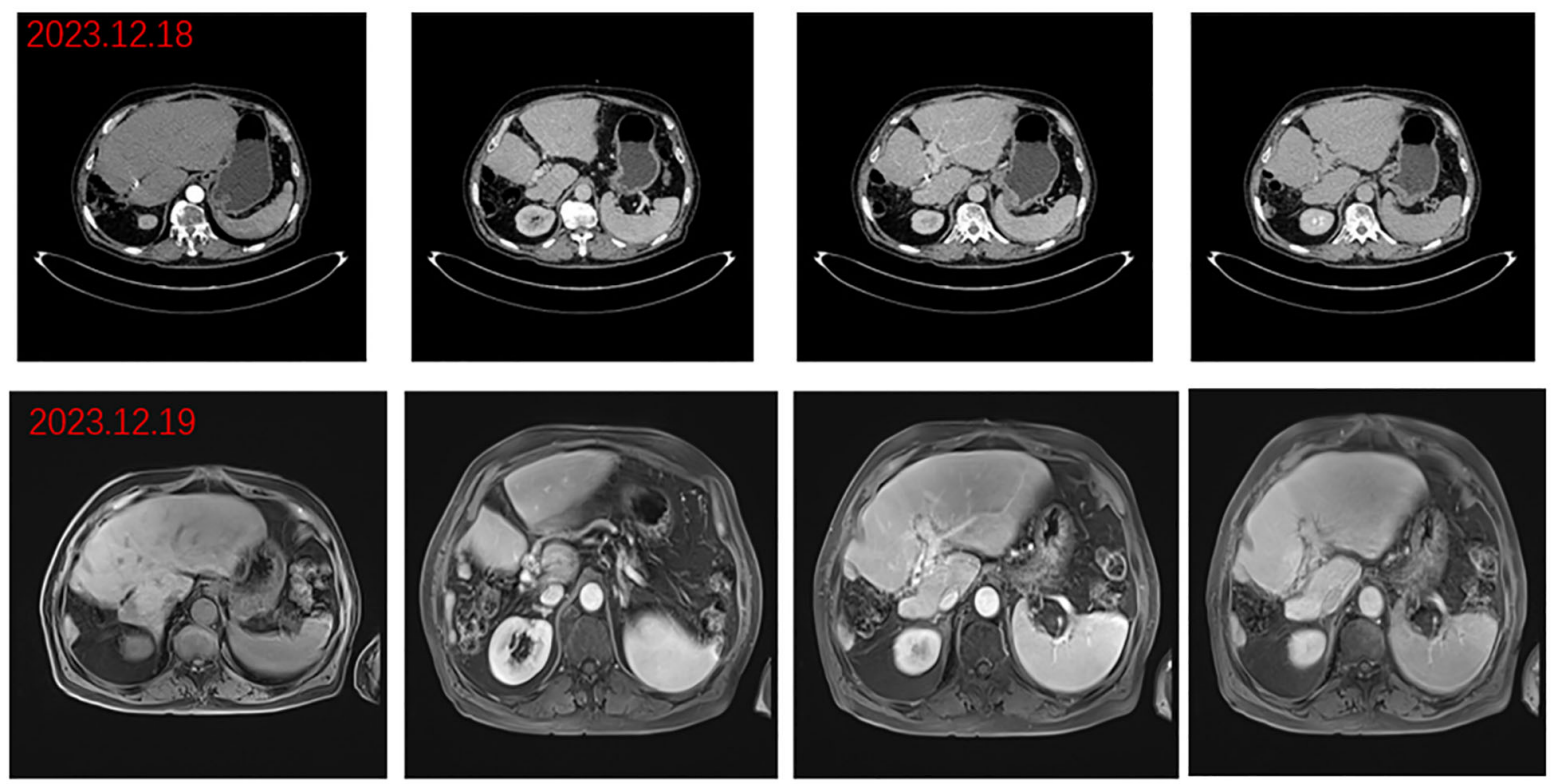
Postoperative images. Arterial, portal, and delayed phase CT: disappearance of portal cancer embolus and resection of right lobe lesion and portal phase, delayed phase abdominal MR.

The patient was a 76-year-old male who presented to Lanzhou University First Hospital on August 5, 2021, with upper abdominal bloating and mild pain persisting for over one month. He reported no other symptoms, and an abdominal ultrasound conducted at another local hospital indicated the presence of a liver mass. His medical history included chronic hepatitis B for over 30 years, hypertension, diabetes, and cataract surgery with intraocular lens implantation. Upon physical examination, the patient exhibited normal skin and mucosal color without jaundice, a flat abdomen devoid of abdominal wall varices, and no remarkable gastrointestinal contour or peristalsis. There was no tenderness upon abdominal palpation, and the liver was palpable below the rib margin. Murphy’s sign was negative, and bowel sounds were noted at a rate of four times per minute. Laboratory tests revealed the following values within normal limits: white blood cell count (WBC: 5.03 × 10^9/L), neutrophil ratio (NEUT%: 68.8%), hemoglobin (HGB: 124 g/L), and platelet count (PLT: 205 × 10^9/L). Tumor markers were elevated, including alpha-fetoprotein (AFP: 289 U/mL), carbohydrate antigen 19-9 (CA 19-9: 109 U/mL), carcinoembryonic antigen (CEA: 1.4 ng/mL), and ferritin (519 ng/mL). Biochemical analysis indicated that aspartate aminotransferase (AST: 67 U/L), alanine aminotransferase (ALT: 216 U/L), total bilirubin (TBIL: 16.4 µmol/L), direct bilirubin (DBIL: 4.8 µmol/L), alkaline phosphatase (ALP: 216 U/L), gamma-glutamyl transferase (GGT: 371 U/L), and glucose (GLU: 6.59 mmol/L) were outside normal ranges. Tests for hepatitis B (2+, 5+) and hepatitis B virus DNA (HBV DNA < 100 IU/mL) were negative. Brain natriuretic peptide (BNP: 143.4 pg/mL) was outside the normal range. The enhanced abdominal CT scan revealed a large mass-like abnormal enhancement in the right lobe of the liver, suggesting hepatocellular carcinoma (HCC) with portal vein thrombosis in the main trunk as well as the left and right branches, cirrhosis, and portal hypertension (including esophageal and gastric fundus varices). Magnetic resonance imaging (MRI) of the liver revealed a large abnormally enhanced mass in the right lobe, suggesting hepatocellular carcinoma (HCC) with intratumoral hemorrhage and portal vein thrombosis in the main trunk as well as the left and right branches; cirrhosis, splenomegaly, a small amount of ascites, liver disease, and gallbladder disease (**Figure 1**). The patient was diagnosed with HCC of the right lobe, post-hepatitis B cirrhosis, grade 3 hypertension (very high risk), and type 2 diabetes, classified as stage IIIa according to the CNLC staging system (**Figure 2**). An initial multidisciplinary team (MDT) discussion resulted in a treatment plan that included hepatic artery infusion chemotherapy (HAIC) treatment (FOLFOX), combined with 200 mg sintilimab and sorafenib 0.4 g bid targeted therapy. The patient was discharged on August 16^th^, 2021. After discharge, oral sorafenib 0.4g bid targeted therapy was prescribed. On the second admission, a second MDT discussion was held to adjust the protocol: HAIC treatment and percutaneous liver puncture for radioactive ^125^I particle implantation, combined with 200 mg sintilimab and sorafenib targeted therapy. After the fifth admission, there was no significant change in the portal vein tumor thrombus. The third MDT was conducted, and on March 29, 2022, “percutaneous hepatic puncture radioactive 125I particle implantation” radiation therapy was performed. Additionally, because the patient could not tolerate bisphosphonates, the treatment was changed to once-daily targeted therapy with 8 mg of lenvatinib. The patient underwent abdominal CT (plain + enhanced) at the sixth admission on June 21, 2022, compared with the previous scan on March 28, 2022 showed a lesion in the right lobe of the liver with little change, along with occlusion of the main portal vein and portal spongiosis, which remained little changed from the previous scan (**Figure 2** **).** After comprehensive consideration of the timing of surgical resection following successful conversion, the decision was made to perform surgical treatment. Subsequently, another preoperative evaluation was conducted, which included gastroscopy, revealing a 0-Ia+IIc type lesion can be seen near the anterior wall of the cardia, with a size of about 3 × 2 cm and chronic atrophic gastritis (**Figure 3A**). After the fourth MDT, the gastric tumor was initially resected via laparoscopy combined with endoscopy under general anesthesia on July 4, 2022 (endoscopic mucosal resection, partial gastrectomy, and D1 lymph node dissection). Due to the patient’s poor cardiopulmonary function during the operation, the laparoscopic surgery was interrupted and converted into an open procedure for right hepatectomy and cholecystectomy (**Figure 3B**). The postoperative gastric histopathology report indicated ectopic hyperplasia of adenoepithelial hyperdifferentiated epithelial endothelium and focal carcinomatous lesions (**Figure 3C**). Two trans arterial chemoembolization (TACE) procedures were performed on September 13, 2022, and April 3, 2023, as adjuvant treatment following hepatic resection, in accordance with the Guidelines for the Diagnosis and Treatment of Primary Hepatocellular Carcinoma (2022 edition). Postoperative abdominal CT and MRI were performed on December 18, 2023, which revealed the disappearance of the portal vein thrombus and widening of the hepatic fissure (**Figure 4**). Remarkably, the patient demonstrated a favorable prognosis, recovering well and remaining free of tumor recurrence or metastasis during over eight months of follow-up.

## 3 Discussion

In China, liver cancer accounts for 75%–85% of malignant tumors in the digestive system (11); however, cholangiocarcinoma stands up to 10%–15% of malignant tumors in digestive system (12). Early stage of liver cancer can be treated with surgery, radiofrequency ablation, and other therapies, with a median survival time of over five years. For surgically resected patients, the five-year survival rate can reach 70%, and the surgical mortality rate is less than 5% (13, 14). Patients with advanced-stage liver cancer and poor prognosis which results from the fact that liver disease cannot undergo radical surgery directly (15). Therefore, conversion therapy for liver cancer is a clinically important issue.

Recently, the widespread application of targeted drugs (e.g., small molecule tyrosine kinase inhibitors (TKIs) and angiogenesis inhibitors) and immune checkpoint inhibitors (ICIs) has ushered in a new era of targeted immunotherapy for liver cancer treatment and conversion therapy. In recent clinical practice, combined targeted immunotherapy and local treatment have facilitated surgical resection for initially unresectable liver cancer patients with expert consensus (16).

According to the guideline of Primary Liver Cancer Diagnosis and Treatment Guidelines (2022 edition), multiple targeted drugs (e.g., donolutamide, lenvatinib, sorafenib, apatinib, bevacizumab, regorafenib, cabozantinib, and ramolutamide) and immunotherapeutic drugs (e.g., tislelizumab, camrelizumab, durvalumab + tremolutamide, and nivolumab + ipilimumab) are recommended for the first-line/second-line treatment of advanced liver cancer (17). Commonly used drug combinations for transformation therapy include Coley’s combination (TKI + ICI) (18–20), T+A (atezolizumab + bevacizumab) (21), and the double D combination (sintilimab + zolbetuximab) (22). In addition, local treatments, such as transcatheter arterial chemoembolization (TACE), hepatic artery infusion chemotherapy (HAIC), radiotherapy, and ablation, are often used in combination with targeted immunosuppressants for conversion therapy. The triple combination of TACE, ICI, and TKI has a higher success rate of surgical resection after HCC conversion therapy and a better prognosis than the combination of TACE and TKI (23, 24).

The timing of surgery should be based on tumor response. The main pathological response (MPR) refers to a significant reduction in the proportion of viable tumor cells to a clinically significant threshold. Pathological complete response refers to the absence of viable tumor cells in the resected specimen after complete evaluation of all sampled areas, including lymph nodes, cancer emboli, and distant metastases. Radiographic evaluation should use the modified RECIST (mRECIST) criteria instead of traditional RECIST v1.1 criteria for evaluating liver lesions’ response to treatment. In addition, the timing of surgery should also consider the safety of the operation, an important aspect to evaluate before transformation resection. This not only requires to assess the necessary safety checks for general liver resection surgery but also to investigate the potential effect of earlier conversion therapy on the liver. When stage IIIa liver cancer is combined with early gastric cancer (EGC), the LECS function can be selected. EGC refers to gastric cancer that invades no deeper than the submucosa layer, regardless of lymph node metastasis (T1 or any N stage). Deng et al. reported that for gastric body type IIa + IIb lesions diagnosed with high-grade intraepithelial neoplasia, ESD was performed (25). For gastric cancer staging, cT1b(SM)N0 is recommended for surgical resection with D1 or D1 + lymph node dissection. In 2012, Nunobe et al. proposed LECS for early gastric cancer. Generally, LECS is easier to perform, has a shorter operation time, and has fewer complications compared to ESD.

Recently, the concept and process of conversion therapy have become increasingly sophisticated (26). Hence, liver cancer patients will be able to receive alternative conversion therapy in the future. However, there remain many clinical issues that require further evaluation.

## Data Availability

The original contributions presented in the study are included in the article/supplementary material. Further inquiries can be directed to the corresponding author/s.
